# High Serum Uric Acid and Increased Risk of Type 2 Diabetes: A Systemic Review and Meta-Analysis of Prospective Cohort Studies

**DOI:** 10.1371/journal.pone.0056864

**Published:** 2013-02-20

**Authors:** Qin Lv, Xian-Fang Meng, Fang-Fang He, Shan Chen, Hua Su, Jing Xiong, Pan Gao, Xiu-Juan Tian, Jian-She Liu, Zhong-Hua Zhu, Kai Huang, Chun Zhang

**Affiliations:** 1 Department of Nephrology, Union Hospital, Tongji Medical College, Huazhong University of Science and Technology, Wuhan, China; 2 Department of Neurobiology, Tongji Medical College, Huazhong University of Science and Technology, Wuhan, China; 3 Department of Cardiovascular Diseases, Union Hospital, Tongji Medical College, Huazhong University of Science and Technology, Wuhan, China; MOE Key Laboratory of Environment and Health, School of Public Health, Tongji Medical College, Huazhong University of Science and Technology, China

## Abstract

**Objective:**

Current evidence suggests high serum uric acid may increase the risk of type 2 diabetes, but the association is still uncertain. The aim of the study was to evaluate the association between serum uric acid and future risk of type 2 diabetes by conducting a meta-analysis of prospective cohort studies.

**Design and Methods:**

We conducted a systematic literature search of the PubMed database through April 2012. Prospective cohort studies were included in meta-analysis that reported the multivariate adjusted relative risks (RRs) and the corresponding 95% confidence intervals (CIs) for the association between serum uric acid and risk of type 2 diabetes. We used both fix-effects and random-effects models to calculate the overall effect estimate. The heterogeneity across studies was tested by both *Q* statistic and *I^2^* statistic. Begg’s funnel plot and Egger’s regression test were used to assess the potential publication bias.

**Results:**

We retrieved 7 eligible articles derived from 8 prospective cohort studies, involving a total of 32016 participants and 2930 incident type 2 diabetes. The combined RR of developing type 2 diabetes for the highest category of serum uric acid level compared with the lowest was 1.56(95% CI, 1.39–1.76). Dose-response analysis showed the risk of type 2 diabetes was increased by 6% per 1 mg/dl increment in serum uric acid level (RR 1.06, 95% CI: 1.04–1.07). The result from each subgroup showed a significant association between serum uric acid and risk of type 2 diabetes. In sensitive analysis, the combined RR was consistent every time omitting any one study. Little evidence of heterogeneity and publication bias was observed.

**Conclusions:**

Our meta-analysis of prospective cohort studies provided strong evidence that high level of serum uric acid is independent of other established risk factors, especially metabolic syndrome components, for developing type 2 diabetes in middle-aged and older people.

## Introduction

Type 2 diabetes is an increasingly important disease globally. New data from IDF showed that there are 336 million people with diabetes in 2011 and this is expected to rise to 552 million by 2030 [1]. It has been suggested that, diabetic epidemic will continue even if the level of obesity remains constant [1], [2]. Thus, identifying risk factors which are responsible for its incidence is urgently required for the prevention of type 2 diabetes.

It is has long been hypothesized that hyperuricemia might be a risk factor for the development of type 2 diabetes, but the casual association between hyperuricemia and type 2 diabetes remains controversial. Since elevated serum uric acid levels are often associated with established type 2 diabetes risk factors, such as alcohol consumption and metabolic syndrome, it is still unclear whether serum uric acid is merely a risk marker or an independent risk factor for diabetes. A previous meta-analysis [3] of 11 combined cohort studies found a significant relationship between elevated serum uric acid level and risk of developing type 2 diabetes, indicating a 17% increment in the risk of diabetes per 1 mg/dl increase in serum uric acid level. Of note, however, the overall effect estimate might be inaccurate in that review because of a statistically significant publication bias, as well as the presence of large heterogeneity across the included studies, which both reduced the validity of the result. Furthermore, 7 of 11 studies were retrospective cohort studies, which had more biases than prospective cohort study design.

Very recently, several well-designed prospective studies [4], [5], [6] provided stronger evidence concerning the relationship between high serum uric acid level and the risk of type 2 diabetes. All these prospective studies adjusted for metabolic syndrome components to validate an independent association between uric acid and diabetes, which was not sufficiently demonstrated previously. Given the above, our goal, therefore, was to evaluate whether serum uric acid was associated with future risk of incident type 2 diabetes independent of established risk factors, especially metabolic syndrome components, by conducting a meta-analysis of prospective cohort studies.

## Design and Methods

### Search Strategy

We reported the meta-analysis according to the recommendations of the Meta-analysis of Observational Studies in Epidemiology (MOOSE) [7] and the Preferred Reporting Items for Systematic Reviews and Meta-analyses (PRISMA) [8] (Checklist S1). We searched the PubMed database for all relevant studies though April 2012 using the following search strategy: (uric acid OR hyperuricemia OR urate) AND diabetes AND (risk factors OR prospective OR prospective study OR cohort OR cohort study OR follow-up OR follow-up study). No language limitations were used. In addition, the reference lists of the retrieved studies were reviewed to integrate the search strategy.

### Study Selection

Studies were included if they met the following criteria: 1) the study design was a prospective cohort study; 2) the exposure of interest was serum uric acid; 3) the outcome of interest was incidence of type 2 diabetes; 4) the relative risk (RR) and the corresponding 95% confidence interval (CI) for the highest compared with the lowest category of serum uric acid levels were reported. Meanwhile, we excluded the studies that were cross-sectional studies, case-control studies, retrospective cohort studies or sub-analysis of randomized controlled trials; or that only reported unadjusted or sex and age adjusted RR; or that reported RR but not 95% CI; or that were duplicated. If the same population was reported in more than one studies, we included the one with the longest follow-up duration and with the most complete data.

### Data Extraction

In each primary study, the serum uric acid levels have been classified into four or five categories to examine the effect of each category on risk of type 2 diabetes. We extracted all the multivariate adjusted RRs and the corresponding 95% CIs based on the highest category of serum uric acid level compared with the lowest, except the ones from the Framingham Heart Study in which the serum uric acid levels were classified into five categories. Instead of the fifth category (the highest category), the fourth category of serum uric acid level was used to compared with the lowest for the effect estimate, since the highest category group from original cohort had a very small number of participants (less than 1% of total), which might result in an insufficient statistically power. In addition, the adjusted RRs selected for analysis were the ones adjusting for the main potential confounders in multivariate analysis. All serum uric acid values in umol/L were converted to mg/dl by dividing by 59.5.

Instead of providing aggregate scores, we assessed the quality of primary studies by reporting the key components of each study design [7]. The retrieved components included last name of the first author, year of publication, country of origin and cohort name, duration of follow-up, participants’ age and sex, number of incident cases and total participants, range of serum uric acid levels, ascertainment of type 2 diabetes, adjusted RR and the corresponding 95% CI, and adjusted confounders in multivariate analysis. Two authors (QL and XFM) independently conducted the literature search, study selection and data extraction. Any disagreements were resolved by discussion.

### Statistical Analysis

We used multivariate adjusted RRs and the corresponding 95% CIs for statistically analysis. The risk ratio or hazard risk in each primary study was directly considered as RR. We used fix-effects model to combine these RRs to get an overall RR, also known as effect estimate. If the heterogeneity across studies was present, a random-effects model would be used. In fact, both models yielded essentially identical results. The heterogeneity across studies was tested by *Q* statistic [9] based on the Chi-square test and a *P* level of less than 0.1 was considered significant. Furthermore, a quantitative measure of the heterogeneity was calculated by *I^2^* statistic [10].

We also conducted a dose-response analysis of the association between serum uric acid and risk of type 2 diabetes based on the following data from individual studies: categories of serum uric acid levels, number of cases and participants, adjusted RR and the corresponding 95% CI. Each RR was transformed into its nature logarithm value (logRR) and its corresponding 95% CI was used to calculate the logRR’s standard error (selogRR). The dose-response relationship was estimated by means of generalized least squares (GLST) [11], [12], which was used for linear trend estimation of summarized dose-response data.

Pre-specified subgroup analyses were performed to evaluate the impact of various factors on the outcome according to mean age (<50 years vs ≥50 years), geographic area (Asians vs non-Asians), and adjustment levels (physical activity vs non-physical activity; hereditary vs non-hereditary; alcohol consumption vs non-alcohol consumption; serum creatinine or non-creatinine; plasma glucose vs non-plasma glucose). Furthermore, we conducted a sensitive analysis to investigate the influence of a single study on the overall effect estimate by omitting one study in each turn.

Potential publication bias was assessed by Begg’s funnel plot and Egger’s regression test [13]. A *P* level of less than 0.1 was considered significant. All data were analyzed using STATA version 12.0 (StataCorp). A *P* level of less than 0.05 was considered statistically significant unless otherwise specified. All *P* values were two-tailed.

## Results

### Literature Search

**Figure 1:**
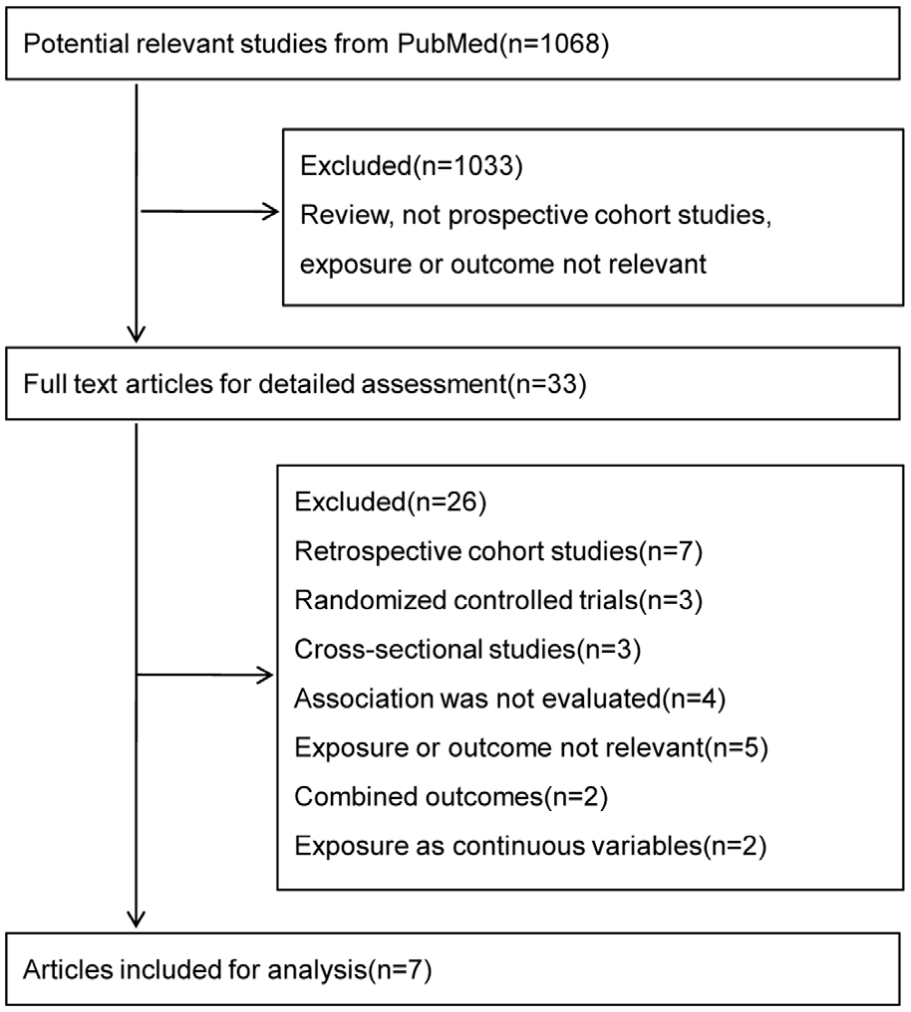
Flowchart of the study selection.

We initially retrieved a total of 1068 citations from the PubMed database and the reference lists the primary studies, of which 1033 citations were excluded after the first screening based on titles and abstracts. Finally, 33 full-text articles were reviewed for detailed assessment, of which 7 eligible articles were included for the meta-analysis. 26 studies were excluded for the following main reasons: 13 studies were not prospective cohort study design, 2 studies [14], [15] reported combined impaired fasting glucose (IFG) and type 2 diabetes as the outcome, and the residual studies were either irrelevant or not evaluate the association between serum uric acid and type 2 diabetes. We further excluded two prospective cohort studies [16], [17] in which serum uric acid levels were treated as continuous variables but not categories, since there is no appropriate statistical method for converting continuous exposure variables into categories to estimate the outcome. A flowchart of the study selection process was presented in Figure 1.

### Study Characteristics

The characteristics of the included studies were presented in Table 1. Our meta-analysis finally included 7 articles derived from 8 prospective cohort studies, involving a total of 32016 participants and 2930 incident type 2 diabetes. The duration of follow-up period ranged from 3.5 years to 28 years, with a median of 11 years. Of these, 3 studies were conducted in Europe, 3 in China and 2 in US. The Framingham Heart Study [6] conducted in US had two independent original and offspring cohort and two studies [18], [19] were performed in men only. All the individual studies were based on general population of predominantly middle-aged and older participants, except one study [5] focusing on the hospital-based hypertensive patients. Participants in all studies were without diabetes at entry and 4 studies [5], [18], [19], [20] of which additionally excluded individuals with cardiovascular disease, cancer or uric acid lowering treatment at entry. All the RRs in each original study were estimated based on the highest compared with the lowest category of serum uric acid level. Most studies adjusted for a wide range of potential confounders of the association between serum uric acid and risk of type 2 diabetes, including age, sex, BMI, blood pressure, other components of metabolic syndrome (HDL cholesterol, triglycerol and plasma glucose) and lifestyle (smoking, alcohol consumption and physical activity), some of them additionally adjusted for hereditary (family history of diabetes) and serum creatinine level (or eGFR), but only one additionally adjusted for insulin (or insulin resistance).

**Table 1:** Characteristics of studies included in the meta-analysis.

Study (ref.)	Location, cohort	Population	Follow-up (years)	Age (mean)	Men%	Case/total
Perry 1995 [19]	British, The Regional HeartStudy (1978–1980)	General without diabetes, cardiovascular disease, other disease or regular drug treatment at entry	12.8	40–59 (50)	100%	194/7577
Taniguchi 2001 [18]	Japan, The Osaka HealthSurvey (1981–1991)	Male workers without diabetes, hypertension, impaired fasting glucose or uric acid lowering medication at entry	5–16	35–61 (42)	100%	454/6356
Chien 2008 [20]	China, Taiwan (1999–2000)	General without diabetes, cardiovascular disease or cancer at entry	9	35–97 (54)	47%	548/2690
Dehghan 2008 [21]	Netherlands, The RotterdamStudy (1991–1995)	General without diabetes at entry	10.1	>55	*	462/4536
Bhole 2010 [6]	US, The Framingham HeartStudy: original cohort (1948)	General without diabetes at entry	28	≥35(45±9)	45%	641/4883
	US, The Framingham HeartStudy: offspring cohort (1971)	General without diabetes at entry	26	≥35(37±10)	48%	491/4292
Viazzi 2011 [5]	Italia, The MAGIC study(1993–1997)	Hospitalized hypertensive patients without diabetes, cardiovascular events, overt nephropathy, gout or allopurinol treatment at entry	11	18–72 (49±10)	56%	42/758
Wang 2011 [4]	China, Shanghai (2005)	General without diabetes at entry	3.5	>40 (62)	36%	98/924

**Study (ref.)**	**Ascertainment of diabetes**	**SUA range (highest vs lowest, mg/dl)**	**Adjusted RR** **(95% CI)**	**Adjusted variables**
Perry 1995 [19]	report	Q5>7 vs Q1<5.1	1.5(0.90–2.5)	Age, BMI, prevalent coronary heart disease (yea/no), physical activity (moderate or inactive), alcohol intake (moderate or occasional), current smoker (yes or never smoked), systolic blood pressure, HDL cholesterol, and heart rate
Taniguchi 2001 [18]	Measure	Q5>6.2 vs Q1<4.2	1.24(0.90–1.71)	Age, BMI, daily alcohol consumption, smoking habits (current, past-, non-smokers), leisure-time physical activity (regular physical activity<once per week), the duration of the walk to work, fasting plasma glucose level, and a parental history of Type 2 diabetes
Chien 2008 [20]	Measure, report	Q5∶7.7 vs Q1∶3.7	1.40(1.02–1.92)	Age, sex, BMI, alcohol intake (nondrinker/current), smoking (yes/no), regular exercise (yes/no), marital status (single, married, or divorced), educational level (<9 years/≥9 years), occupation (no work, manual work, or professional), and family history of diabetes (yes/no), and metabolic syndrome
Chien 2008 [20]	Measure, report	Q5∶7.7 vs Q1∶3.7	1.40(1.02–1.92)	Age, sex, BMI, alcohol intake (nondrinker/current), smoking (yes/no), regular exercise (yes/no), marital status (single, married, or divorced), educational level (<9 years/≥9 years), occupation (no work, manual work, or professional), and family history of diabetes (yes/no), and metabolic syndrome
Dehghan 2008 [21]	Measure, report	Q4>6.2 vs Q1<4.5	1.68(1.22–2.30)	Age, sex, BMI, waist circumference, systolic blood pressure, diastolic blood pressure, and HDL cholesterol
Bhole 2010 [6]	Measure, report	Q4>7 vs Q1<5	1.72(1.21–2.44)	Age, sex, BMI, physical activity level, alcohol consumption, smoking, hypertension, blood glucose level, blood cholesterol level, creatinine level, and triglyceride level
	Measure, report	Q4>7 vs Q1<5	1.71(1.24–2.36)	Age, sex, BMI, physical activity level, alcohol consumption, smoking, hypertension, blood glucose level, blood cholesterol level, creatinine level, and triglyceride level
	Measure, report	Q4>7 vs Q1<5	1.71(1.24–2.36)	Age, sex, BMI, physical activity level, alcohol consumption, smoking, hypertension, blood glucose level, blood cholesterol level, creatinine level, and triglyceride level
Viazzi 2011 [5]	Report	men: Q5>7 vs Q1* Women: Q5>5.3 vs Q1*	2.78(1.35–5.7)	Age, sex, BMI, eGFR, components of metabolic syndrome, and metabolic syndrome as a whole
Wang 2011 [4]	Measure, report	Q4∶8.2 vs Q1∶3.1	1.57(1.24–1.99)	Age, sex, BMI, family history of diabetes, smoking, alcohol drinking, systolic blood pressure, diastolic blood pressure, HDL cholesterol, triglycerides, total cholesterol, fasting plasma glucose, fasting insulin, and serum creatinine

Measure = measurement of plasma glucose levels, report = reports from participants or physicians of diagnosis of diabetes, use of anti-diabetic medication and so on, Q = quintiles or quartiles, BMI = body mass index, eGFR = estimated glomerular filtration rate, HDL = high density lipoprotein,

* = not reported.

### Main Analysis

**Figure 2:**
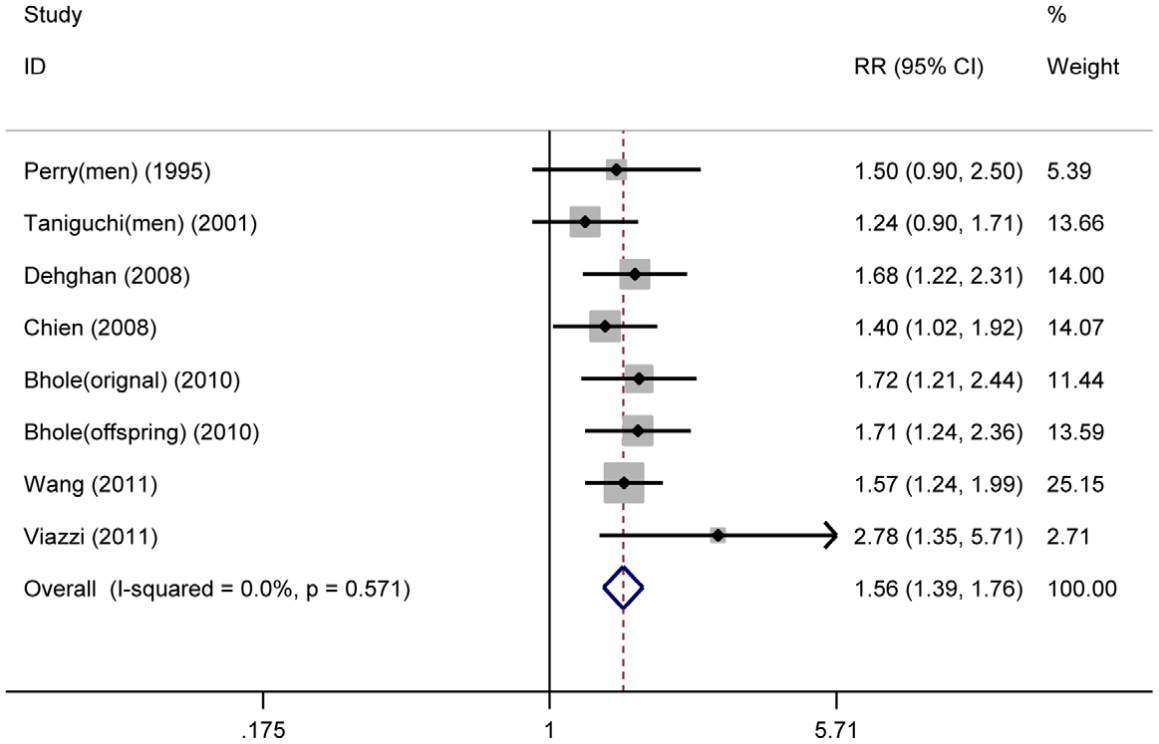
Serum uric acid and risk of incident type 2 diabetes. Fix-effects model analysis for the overall RR (1.56, 95% CI = 1.39–1.76) of incident type 2 diabetes for the highest compared with the lowest category of serum uric acid level. No evidence of heterogeneity across studies was found (*I^2^* = 0.0%, *P* = 0.571). The square sizes are proportional to the weight of each study in the meta-analysis; the horizontal lines represent 95% CIs; the diamond represents the overall RR with its 95% CI.

The multivariate adjusted RR for each study and the combined RR were presented in figure 2. The combined RR of incident type 2 diabetes for the highest category of serum uric acid level compared with the lowest was 1.56 (95% CI, 1.39–1.76). No significant heterogeneity across studies was found (*I^2^* = 0.0%, *P* = 0.571). Apart from using fix-effects model, we also used random-effects model to calculate the pooled effect size. The identical results were observed using both models.

### Dose-response Analysis

Six cohort studies [4], [6], [18], [20], [21] were eligible to had required data for dose-response analysis. The result showed each 1 mg/dl increment in serum uric acid level was significantly associated with 6% increase in the risk of type 2 diabetes (RR 1.06, 95% CI: 1.04–1.07). No evidence of significant heterogeneity was observed between these studies (*I^2^* = 24%, *P* = 0.24).

### Subgroup Analysis and Sensitive Analysis

Table 2 presented the results of subgroup analysis according to mean age, geographic area and various adjusted variables, including physical activity, hereditary, alcohol consumption, serum creatinine level and plasma glucose level. The result from each subgroup showed significant positive association between serum uric acid and risk of type 2 diabetes. Little evidence of heterogeneity was observed in any subgroup.

**Table 2:** Subgroup analysis for assessing the effect of various variables.

Group	Number of studies	RR(95% CI)	P for heterogeneity	I^2^
**Total**	8	1.56(1.39,1.76)	0.571	0.0%
**Mean age**				
** <50 y**	4	1.59(1.31,1.91)	0.171	40.1%
** ≥50 y**	4	1.55(1.32,1.80)	0.88	0.0%
**Geographic area**				
** Asian**	3	1.43(1.22,1.69)	0.503	0.0%
** Non-Asian**	5	1.73(1.45,2.05)	0.735	0.0%
**Adjustment levels**				
** Physical activity**	5	1.49(1.28–1.75)	0.6	0.0%
** Non-physical activity**	3	1.67(1.39,2.00)	0.335	8.5%
** Hereditary**	3	1.43(1.22,1.69)	0.503	0.0%
** Non-hereditary**	5	1.73(1.45,2.05)	0.735	0.0%
** Alcohol intake**	6	1.52(1.33,1.73)	0.72	0.0%
** Non-alcohol intake**	2	1.82(1.36,2.44)	0.21	36.5%
** Creatinine**	4	1.69(1.43,1.98)	0.528	0.0%
** Non-creatinine**	4	1.44(1.21,1.71)	0.616	0.0%
** Plasma glucose**	6	1.55(1.36,1.77)	0.359	8.9%
** Non-plasma glucose**	2	1.63(1.24,2.13)	0.712	0.0%

The result from sensitive analysis showed the remaining studies yielded consistent results every time omitting any one study. The range of the combined RRs from 1.54 (95% CI, 1.36–1.74) to 1.62 (95% CI, 1.43–1.84) was narrow.

### Publication Bias

The Begg’s funnel plot was presented essentially symmetrical. Little evidence of publication bias was found using Egger’s regression test (*P* = 0.246).

## Discussion

In the meta-analysis derived from 8 prospective cohort studies, we found that high level of serum uric acid was associated with increased risk of developing type 2 diabetes in middle-aged and older people. For each 1 mg/dl increment in serum uric acid level, there was a 6% increase in the risk of incident type 2 diabetes. Importantly, the relationship between serum uric acid and incident diabetes was independent of other established risk factors of type 2 diabetes, especially metabolic syndrome components, which strongly supported high serum uric acid as a causal factor of type 2 diabetes.

A previous review from Kodama [3] showed a significant impact of serum uric acid on diabetes incidence. In comparison with this review, however, our study had some important strengths. For example, our meta-analysis had rigorous methods and included relatively high-quality primary studies which all used a prospective cohort study design. In addition, our results showed that there was no significant evidence of heterogeneity and publication bias. More importantly, all primary studies have adjusted for sufficient metabolic syndrome components (defined according to National Cholesterol Education Program Adult Treatment Panel III), but the combined effect estimate remained significant. Overall, our meta-analysis provided stronger evidence to demonstrate the independent contribution of serum uric acid to future risk of type 2 diabetes.

Heterogeneity is a major concern about meta-analysis. However, no evidence of heterogeneity was observed throughout our study. This might be attributed to these facts as follows: all individual studies used a prospective cohort design and adjusted for potential major confounders of type 2 diabetes risk, all but one [4] had a long enough follow-up more than nine years, all had a relatively large number of subjects, and all were conducted based on predominantly middle-aged and older participants without diabetes at entry. Apparently, these aspects above also indicated relatively high-quality characteristics of included studies. In addition to absence of heterogeneity across studies, our results suggested there was no significant publication bias, an important indicator to assess the bias of meta-analysis, which further enhanced the validity of our results.

In the subgroup analysis, all the results were statistically significant and had no evidence of heterogeneity, indicating that the significant relationship between serum uric acid and the development of type 2 diabetes was not affected by various stratified factors. However, the association seemed to be stronger in subgroup of mean age <50 years than in ≥50 years, as well as in Western countries than in Asian countries, whereas the effect was attenuated but still significant after adjustment for physical activity, hereditary, alcohol consumption or plasma glucose level respectively, except after adjustment for serum creatinine level. The existence of stronger effect after adjustment for creatinine level might be due to the fact that most of these studies were conducted in Western countries which had a stronger association between serum uric acid and type 2 diabetes. Similarly, the results from sensitive analysis were significant and robust, suggesting that the overall effect estimate was not driven by any single study. Furthermore, we identified a significant dose-response relationship between serum uric acid and incident type 2 diabetes, showing a 6% increase in the risk of diabetes per 1 mg/dl increment in serum uric acid level, which further strengthened the cause-effect association.

Several underlying mechanisms might be involved in the association between hyperuricemia and the development of type 2 diabetes. For example, recent animal studies showed fructose-induced hyperuricemia play a pathogenic role in metabolic syndrome, and the conditions were improved by decreasing uric acid levels [22], [23]. Hyperuricemia has been shown to induce endothelial dysfunction and to reduce the production of nitric oxide [24], [25]. Nitric oxide reduction could lower insulin-stimulated glucose intake in skeletal muscle, which contributes to insulin resistance and thus diabetes. In addition, hyperuricemia is associated with oxidative stress [26], [27], which plays an important role in the pathogenesis of type 2 diabetes. These experimental evidence supports serum uric acid as a causal factor of diabetes.

Several limitations of our study should be considered. First, although 8 prospective studies included in our meta-analysis had a larger number than an individual study, the sample size might be not large enough. In the current meta-analysis, we excluded 7 retrospective cohort studies based on the following reasons. Firstly, retrospective cohort study design had a relatively low quality of evidence due to having more biases than prospective study design. In addition, although odds ratio (OR) used in a retrospective cohort study could approximate RR, if the outcome of interest was relatively rare (commonly less than 5% [28]), most of these retrospective studies, however, showed that the newly onset diabetes were common (cumulative incidence approximately 10%), resulting in the fact that the OR might overestimate the real relative risk (RR). Finally, exclusion of retrospective studies was also due to the fact that these OR values were estimated based on serum uric acid either as a categorical variable or as a continuous variable (per 1 mg/dl or per 1 SD), which will make it difficult to combine the various OR values.

The second limitation of the current study is that we were unable to stratify these individual studies by the gender because a limited sex-specific study data was available. Previous studies [29], [30] suggested serum uric acid was more strongly associated with cardiovascular disease in women than in men. In our study, four individual studies [4], [5], [6], [21] mentioned high serum uric acid significantly predict the risk of type 2 diabetes in both sexes. However, another study [4] in Shanghai showed the effect of serum uric acid was stronger in men, whereas the association was stronger in women in two other studies [5], [21]. In contrast to these findings, two studies [18], [19] conducted in men found a non-significant association between serum uric acid and diabetes. Therefore, whether the association between serum uric acid and the risk of diabetes is affected by sex difference still needs further more sex-specific studies.

Additionally, although all included studies adjusted for a wide range of potential confounders for risk of incident diabetes, several residual variables including unmeasured (such as dietary factors) and unknown confounders might contribute to the observed association. For instance, high intake of purine-rich food [31] and fructose [32] may induce the development of hyperuricemia. Furthermore, diet is widely believed to play an important role in the development of type 2 diabetes and thus may confound the association between uric acid and diabetes. Of note, none of our included primary studies adjusted for dietary factors in multivariate analysis and further studies related to diet will be needed to validate the relationship of serum uric acid with type 2 diabetes. Finally, our study demonstrated the significant association of serum uric acid with type 2 diabetes was predominantly based on middle-aged and older participants. Interestingly, a recent observational study [33] in adolescents showed a significant association between serum uric acid and the development of hypertension. However, whether there is a significant association of serum uric acid with type 2 diabetes in children and adolescents is still unclear.

In conclusion, our meta-analysis of prospective cohort studies provided strong evidence that high level of serum uric acid is independent of other established risk factors, especially metabolic syndrome components, for developing type 2 diabetes in middle-aged and older people. Our findings have important clinical implications. Given the fact that type 2 diabetes has been a growing public health burden across the world and hyperuricemia is very common in the general population [34], [35], early identification of hyperuricemia will be of importance. Moreover, serum uric acid levels can be easily measured and hyperuricemia is modifiable by medication. Therefore, controlling hyperuricemia might be a promising strategy for the prevention of type 2 diabetes.

## Supporting Information

Checklist S1 **PRISMA Checklist for systematic review and meta-analysis.**
(DOC)
